# Patient preferences for the design of a pharmacy-based colorectal cancer screening program

**DOI:** 10.1007/s10552-023-01687-x

**Published:** 2023-04-19

**Authors:** Alison T. Brenner, Austin R. Waters, Mary Wangen, Catherine Rohweder, Olufeyisayo Odebunmi, Macary Weck Marciniak, Renée M. Ferrari, Stephanie B. Wheeler, Parth D. Shah

**Affiliations:** 1 Division of General Medicine and Clinical Epidemiology, Department of Medicine, University of North Carolina School of Medicine, Chapel Hill, NC USA; 2 Lineberger Comprehensive Cancer Center, University of North Carolina at Chapel Hill, Chapel Hill, NC USA; 3 Department of Health Policy and Management, University of North Carolina at Chapel Hill, Chapel Hill, NC USA; 4 Center for Health Promotion and Disease Prevention, University of North Carolina at Chapel Hill, Chapel Hill, NC USA; 5 Eshelman School of Pharmacy, University of North Carolina at Chapel Hill, Chapel Hill, NC USA; 6 Public Health Sciences Division, Fred Hutchinson Cancer Center, Seattle, WA USA; 7 Department of Maternal and Child Health, University of North Carolina at Chapel Hill, Chapel Hill, NC, USA

**Keywords:** Colorectal cancer, Pharmacy, Stool-based testing, Screening

## Abstract

**Purpose:**

To assess preferences for design of a pharmacy-based colorectal cancer (CRC) screening program (PharmFIT™) among screening-eligible adults in the United States (US) and explore the impact of rurality on pharmacy use patterns (e.g., pharmacy type, prescription pick-up preference, service quality rating).

**Methods:**

We conducted a national online survey of non-institutionalized US adults through panels managed by Qualtrics, a survey research company. A total of 1,045 adults (response rate 62%) completed the survey between March and April 2021. Sampling quotas matched respondents to the 2010 US Census and oversampled rural residents. We assessed pharmacy use patterns by rurality and design preferences for learning about PharmFIT™; receiving a FIT kit from a pharmacy; and completing and returning the FIT kit.

**Results:**

Pharmacy use patterns varied, with some notable differences across rurality. Rural respondents used local, independently owned pharmacies more than non-rural respondents (20.4%, 6.3%, *p* < 0.001) and rated pharmacy service quality higher than non-rural respondents. Non-rural respondents preferred digital communication to learn about PharmFIT™ (36% vs 47%; *p* < 0.001) as well as digital FIT counseling (41% vs 49%; *p* = 0.02) more frequently than rural participants. Preferences for receiving and returning FITs were associated with pharmacy use patterns: respondents who pick up prescriptions in-person preferred to get their FIT (OR 7.7; 5.3–11.2) and return it in-person at the pharmacy (OR 1.7; 1.1–2.4).

**Conclusion:**

Pharmacies are highly accessible and could be useful for expanding access to CRC screening services. Local context and pharmacy use patterns should be considered in the design and implementation of PharmFIT™.

**Supplementary Information:**

The online version contains supplementary material available at 10.1007/s10552-023-01687-x.

## Introduction

To maximize the benefits of colorectal cancer (CRC) screening for underserved populations, public health professionals need to reach more eligible adults using more equitable approaches. In this case, we consider *equity* to be improving CRC screening coverage for populations with poorer access to primary care where screening normally occurs [1–3], such as  populations in rural regions and others that live in medically underserved areas or populations (MUA, MUP) or health professional shortage areas [4]. In these communities, alternative healthcare delivery settings that may be more plentiful or accessible should be considered for delivery of CRC screening tests that do not require a primary care visit or direct physician involvement.

Fecal immunochemical testing (FIT) is one method of CRC screening that can occur outside a primary care visit. FITs can be provided with or without a physician’s order and processed at a community medical laboratory; the results of the FIT can be transmitted to a primary care provider by any secure method (e.g., fax). FITs have some advantages over other screening modalities: they are less expensive than screening colonoscopies and have demonstrated a reduction in CRC mortality when compared to no screening. [5, 6] Innovative strategies for increasing the use of FIT, such as combining the test with flu shots (Flu-FIT) [7, 8] and mailing the kits to patients’ homes [9–12], have resulted in higher uptake of screening. Furthermore, FIT outreach programs improve access to screening tests for rural residents and patients with low income [10, 13].

A novel approach to increasing access to FIT is a pharmacy-based model where patients receive CRC screening at local community pharmacies. Pharmacies are ideally suited for making screening available to adults who are otherwise missed in the traditional healthcare setting. First, pharmacies are highly accessible: 97% of the U.S. population lives within 10 miles of a pharmacy [14] and rural Medicare beneficiaries visit pharmacies nearly three times more often than their primary care providers [15]. Second, community pharmacies increasingly serve as a source of preventive services, such as immunizations, wellness coaching, and diabetes self-management [16]. Third, FITs are suitable for distributing to patients at pharmacies because they are inexpensive, home-based tests that can be mailed back to a lab or delivered in-person after they are completed. Pharmacies already have practices and systems in place to counsel patients, send reminders, adjudicate insurance plans in real time, contract with labs, and communicate with other healthcare providers. Little research conducted in the U.S. has explored pharmacy-based CRC screening models [17, 18]. To address this gap, the purpose of this study was to assess patient preferences for the design of a pharmacy-based FIT distribution program we call PharmFIT™. The goal of our formative work was to determine specific needs and preferences for a FIT program targeting rural and medically underserved populations. Findings come from the PharmFIT™ Patient Survey, a national online survey of US adults.

## Methods

### Participants and procedures

Survey participants were non-institutionalized U.S. adults and members of a market research panel maintained and operated by Qualtrics, a survey research company [19]. The panel was created using various suppliers that use a diverse set of recruitment methodologies [20]. The use of multiple sampling sources ensures that the overall sampling frame is not reliant on any specific demographic or segment of the U.S. population [20]. A comparison of online samples of adults from Facebook, MTurk, and Qualtrics found that Qualtrics recruited samples came the closest to matching the demographic composition of a national probability sample from a gold-standard survey in the US [21, 22].

5,537 panel members responded to the survey invitation and completed the eligibility screener. Eligible participants were U.S. adults aged 45–75 [23], of low to average risk of developing colorectal cancer (i.e., no personal/family history of polyps, colorectal cancer, or inflammatory bowel disease), and willing to use FIT for future CRC screening. Sampling quotas were applied to ensure a match to the 2010 U.S. Census for racial, ethnic, and sex groups, and to oversample rural residents to represent approximately one in three respondents. A total of 1,045 adults were eligible, provided informed consent, and completed the survey between March and April 2021. After accounting for panel members of unknown eligibility who accessed the survey but were excluded by Qualtrics because of over quotas (*n* = 2,085), ineligible panel members (*n* = 2,128), and excluding participants whose survey responses were flagged for data quality issues (*n* = 229) or for speeding (defined by the survey company as answering questions too quickly to have comprehended question) (*n* = 50), the survey response rate was 62% (*N* = 1,042), calculated using the American Association for Public Opinion Research Response Rate 4 [24]. A detailed explanation of our response rate calculation is included in an online appendix. The analytic sample of survey responders came from all 50 states, Washington D.C., and Puerto Rico. Responders were an average of 59.5 years old, half female, and primarily non-Hispanic White. The most common type of insurance reported was private followed by Medicare and Medicaid. The majority of participants had household incomes of less than $60,000 and reported having had a recent CRC screening. Demographic characteristics are reported in Table 1.

**Table 1:** Participant demographic characteristics (*n* = 1,045)

	*n* or mean (% or SD)
Age	59.5 (8.6)
Gender	
Male	523 (50.1)
Female	522 (49.9)
Race	
White	770 (73.7)
Black	134 (12.8)
Asian	68 (6.5)
Multiracial or other race	73 (7.0)
Ethnicity	
Hispanic or latino/a	126 (12.1)
Not hispanic or latino/a	919 (87.9)
Screening history	
Recent screening	689 (65.9)
No recent screening	356 (34.1)
Insurance status	
Private	407 (39.4)
Medicare	331 (32.1)
Medicaid	163 (15.8)
VA/TRICARE/IHS/Other	71 (6.9)
Uninsured	60 (5.8)
Rurality	
Rural	314 (30.1)
Not rural	731 (70.0)
Travel time to nearest healthcare provider (minutes)*	18.7 (14.2)
Education	
High school education or less	217 (20.8)
Some college	408 (39.0)
College degree	259 (24.8)
Graduate education or higher	161 (15.4)
Household income	
< $20,000	156 (14.9)
$20,000–$39,999	247 (23.6)
$40,000–$59,999	219 (21.0)
$60,000–$79,999	151 (14.5)
$80,000–$99,999	89 (8.5)
$100,000 +	183 (17.5)
General health status	
Excellent	77 (7.4)
Very good	301 (28.8)
Good	443 (42.4)
Fair	190 (18.2)
Poor	34 (3.3)

Screening history was collapsed from a question asking which screening participants received most recently. If any screening was selected participate was classified as having recent screening. If a participant selected more than one insurance including Medicaid or Medicare, they were coded in Medicaid or Medicare rather than the private insurer/supplemental

*Range: 0–120, IQR: 10–25

The institutional review boards at the University of North Carolina at Chapel Hill (IRB#18-1337) approved the study protocol.

### Measures

The PharmFIT™ Patient Survey was developed by our research team comprising health services researchers, clinicians (e.g., pharmacists, physicians), and other research staff. The survey questionnaire covered nine different topics about the survey responder: CRC screening experience; Healthcare utilization patterns and their healthcare provider; the pharmacy they typically uses for prescription medications [25]; PharmFIT™ program design; Diffusion of Innovation; Willingness to use PharmFIT™; Follow-up care; Telemedicine; and Demographic characteristics. Survey items were newly developed or adapted from other sources [26]. Our study focused on a subset of these topics.

We conducted six cognitive interviews with survey-eligible adults to ensure participants understood survey items in the way the study team intended. Qualtrics pretested the survey with 119 eligible participants on their panel to ensure that the questionnaire was programmed correctly. The entire survey instrument can be accessed online here: https://dataverse.unc.edu/dataverse/cpcrn-4cnc-pharmfit.

#### PharmFIT™ program design

**Fig. 1:**
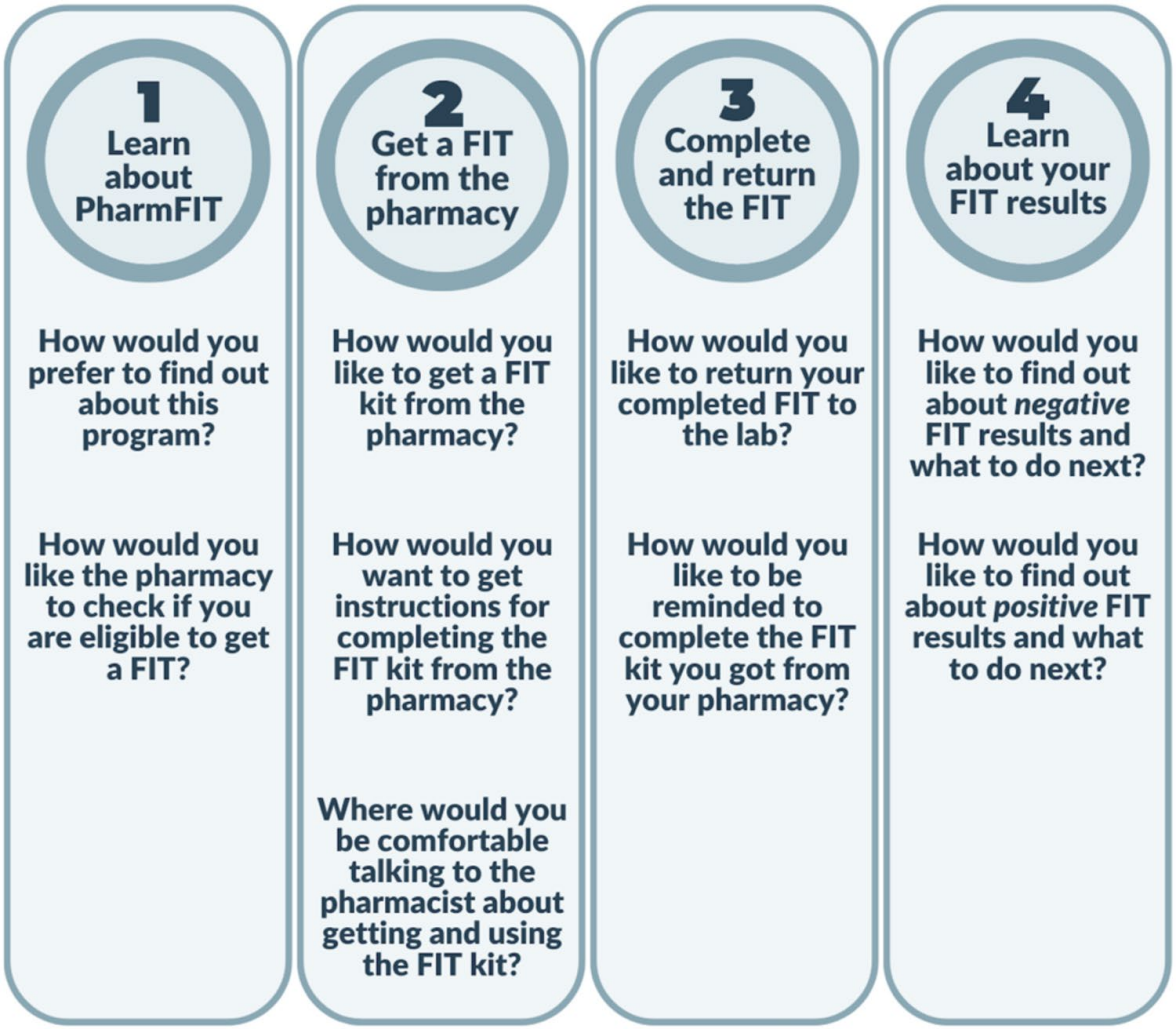
Step of the Pharm﻿^FIT^ Program and Corresponding Survey Design Questions. Step 4 is not analyzed in this manuscript

Eleven multiple choice questions assessed participants’ preferences for using the PharmFIT™ program. The PharmFIT™ program has 4 steps (Fig. 1): (1) learning about the PharmFIT™ program, (2) getting the FIT kit from the pharmacy, (3) completing the FIT kit, and (4) learning about FIT kit results and follow-up. The analysis presented here focuses on steps 1–3 of the PharmFIT™ program. Step 4 will be presented in a separate report. In Step 1, the survey assessed how participants would like to learn about PharmFIT™ and preferences on FIT eligibility determination. In Step 2, the survey assessed participants’ preferences on how they would like the FIT kit distributed and preferences on pharmacist delivered FIT kit counseling. In Step 3, the survey assessed how participants would like to return the completed FIT kit and preferences on how they would like to be reminded to return their kit. We selected FIT kit *pick-up* and *return* preferences as our main outcomes for this study since distributing and getting back FITs are central processes of any FIT distribution program. Since these two survey items used a “check all that apply” response option, we created 5 dichotomous dummy variables based on each response option. For FIT pick-up preferences, the first dummy variable indicated “in-person at the pharmacy” (1) versus not (0) and the second variable indicated “mailed or delivered” (1) versus not (0). For FIT return preferences, the first dummy variable indicated “in-person to pharmacy” (1) versus not (0), the second variable indicated “in-person to the primary care provider” (1) versus not (0), and the third variable indicated “mailed directly to lab” (1) versus “not” (0).

#### Past experiences with pharmacy services

Ten multiple choice questions asked about respondents’ past experiences with pharmacy services. The survey assessed how they received their prescription medication from their pharmacy (e.g., pick-up, mail), what type of pharmacy they use (e.g., retail, clinic), how often they go to the pharmacy for their prescription medications, and what types of non-dispensing clinical services they have used at their pharmacy (e.g., flu shots). The survey also assessed participants’ perceptions about service quality at the pharmacy including familiarity, sympathy, responsiveness, personal attention, safety, and trust. These items had a 5-point response scale that ranged from “strongly disagree” (0) to “strongly agree” (5). Service quality indicator questions were combined into a scale, the pharmacy service quality scale, from 0 to 30 which exhibited high internal consistency (Cronbach alpha = 0.91).

#### Demographic characteristics

The survey also assessed participants’ demographic characteristics including their gender, educational attainment, health insurance, household income, race, ethnicity, rurality, general health status, recent CRC screening, and travel time to the nearest healthcare provider in minutes. Health insurance type was recoded into five categories: Private, Medicare, Medicaid, VA/TRICARE/IHS/Other, and Uninsured. Medicaid and Medicare dominant coding were applied to individuals who reported multiple insurance types. Rurality was classified using RUCC codes mapped to respondent zip codes [27]. Household income was recoded to increments of twenty thousand dollars. Recent CRC screening was recoded as having screening of any type (1) versus not having been screened or not knowing (0).

### Statistical analyses

#### Rural and Urban differences in pharmacy use and PharmFIT™ design preferences

We evaluated whether survey participants’ pharmacy use and PharmFIT™ design preferences varied by rurality. Chi-squared tests were used to identify differences in method of prescription pick-up, type of pharmacy, frequency of visiting the pharmacy, and clinical services available by rurality (Table 2). Kruskal–Wallis tests were used to identify differences in travel time to the nearest healthcare provider and the pharmacy service quality scale (Table 2). Seven PharmFIT™ design items assessing preferences around Steps 1, 2, and 3 were first stratified by rurality and described using means and proportions and visualized using bar charts.

**Table 2:** Rural and urban differences in patients’ pharmacy experiences (*n* = 1,045)

	Not rural	Rural	
	*n* or Mean (% or SD)	*n* or Mean (% or SD)	*p*
Travel time to nearest healthcare provider (Minutes)	18.7 (0.5)	18.8 (0.9)	0.06
How do you usually get your prescription medications from a pharmacy?			
Pick-up at a pharmacy	563 (77.0)	257 (81.9)	0.08
Mailed to my home from a pharmacy	168 (23.0)	57 (18.2)	
Think about the pharmacy you go to most often for prescriptions, over-the-counter medications, or other healthcare needs. This pharmacy is …			
A retail chain pharmacy (like CVS or Walgreens)	391 (53.5)	124 (39.5)	< 0.001
A pharmacy in a grocery store (like Kroger or Albertsons/Safeway)	116 (15.9)	48 (15.3)	
A pharmacy in a department store or wholesaler (like Walmart or Costco)	107 (14.6)	59 (18.8)	
A pharmacy in a clinic or hospital where you receive medical care	71 (9.7)	19 (6.1)	
A local independently owned pharmacy	46 (6.3)	64 (20.4)	
Think about a typical year. About how often did you visit this pharmacy for healthcare needs like medications in a year?			
0–1 times	158 (21.6)	57 (18.2)	0.08
2–5 times	281 (38.4)	107 (34.1)	
6–9 times	88 (12.0)	38 (12.1)	
10 or more times	204 (27.9)	112 (35.7)	
What types of healthcare services beyond prescription filling have you used at this pharmacy?			
Vaccination	278 (38.0)	111 (35.6)	0.41
Point of care testing	51 (7.0)	15 (4.8)	0.18
Chronic disease management	38 (5.2)	17 (5.4)	0.90
Urgent care	37 (5.1)	10 (3.2)	0.18
Other services	42 (5.8)	22 (7.0)	0.44
Pharmacy service quality indicators			
Familiar with pharmacy team	3.2 (0.05)	3.5 (0.07)	< 0.001
Pharmacy team is sympathetic and reassuring	3.7 (0.04)	4.0 (0.05)	0.001
Pharmacy team promptly responds	3.9 (0.04)	4.1 (0.05)	0.004
Pharmacy gives me personal attention	3.7 (0.04)	4.0 (0.05)	< 0.001
Trust in pharmacy team	4.1 (0.03)	4.3 (0.05)	0.002
Feel safe with pharmacy team	4.1 (0.03)	4.3 (0.05)	0.003

Pharmacy service quality indicators were run as continuous and assessed using Kruskal–Wallis tests. Note about check all that apply and not displaying the nos

#### Correlates of FIT pick-up and return

We used multivariable logistic regression models to evaluate preferences for FIT *pick-up* (2 models; Table 3) and *return* (3 models, Table 4). All models included the primary predictor of interest—rurality—and controlled for pharmacy service quality, method used to pick up prescriptions, type of pharmacy, travel time to nearest pharmacy in minutes, age, gender, race, ethnicity, screening history, insurance status, educational attainment, household income, and general health status. Pairwise correlations were checked between all independent variables prior to model building with a preset model exclusion threshold correlation coefficient = 0.05. Independent variables were selected a priori based on correlates of CRC screening in the literature [28, 29]. Further, as a sensitivity analysis, pharmacy-specific variables such as pharmacy quality scale, type of pharmacy, and method used to pick up prescriptions were removed from the model to test impact on demographic characteristic estimates. Estimates in models with and without pharmacy related questions were very similar; therefore, models with pharmacy questions are reported. We used Stata 15.1 (Stata Corp, College Station, TX) for data cleaning and analyses. All statistical tests were 2-tailed with a critical *α* = 0.05.

**Table 3:** Correlates of FIT Kit Pick-up preferences

	In-Person vs. Not (Ref.)		Mail vs. Not (Ref.)	
	*n*/*N* or Mean (SD)	OR (CI_95%_)	*n*/*N* or Mean (SD)	OR (CI_95%_)
Rurality				
Not rural	529/731	Ref	345/731	Ref
Rural	235/314	1.0 (0.7–1.4)	108/314	**0.7 (0.5–0.9)**
Pharmacy quality scale	17.6 (4.8)	**1.1 (1.0–1.1)**	16.7 (5.1)	1.0 (0.9–1.0)
Usual method used to pick up prescriptions				
Mailed by pharmacy	90/225	Ref	160/225	Ref
In-person	674/820	**7.7 (5.3–11.2)**	293/820	**0.2 (0.1–0.3)**
Type of pharmacy most frequented				
A pharmacy in a clinic/hospital	43/90	Ref	62/90	Ref
A retail chain pharmacy	401/515	**3.0 (1.7–5.4)**	214/515	**0.4 (0.2–0.6)**
A pharmacy in a grocery store	123/164	2.0 (0.9–3.8)	64/164	**0.4 (0.2–0.8)**
A pharmacy in a department store or wholesaler	119/166	1.9 (0.9–3.7)	74/166	**0.5 (0.3–0.9)**
A local independently owned pharmacy	78/110	**2.5 (1.2–5.2)**	39/110	**0.3 (0.1–0.6)**
Travel time to nearest provider	18.5 (14.9)	1.0 (0.9–1.0)	19.5 (14.1)	1.0 (0.9–1.0)
Age				
45–64	514/696	Ref	318/696	Ref
65–75	250/349	1.4 (0.9–2.1)	135/349	0.6 (0.9–1.0)
Gender				
Male	365/523	Ref	241/523	Ref
Female	399/522	1.4 (0.9–2.0)	212/522	0.8 (0.9–1.0)
Race				
White	575/770	Ref	306/770	Ref
Black	96/134	0.7 (0.5–1.2)	69/134	**1.5 (1.0–2.3)**
Asian	50/68	1.1 (0.6–2.1)	32/68	1.1 (.06–1.9)
Multiracial or other race	43/73	**0.5 (0.3–0.9)**	46/73	**2.3 (1.3–4.0)**
Ethnicity				
Non-hispanic or latino/a	675/919	Ref	390/919	Ref
Hispanic or latino/a	89/126	1.0 (0.6–1.6)	63/126	1.1 (0.7–1.7)
Screening history				
Recent screening	498/689	Ref	297/689	Ref
No recent screening	266/356	0.8 (0.6–1.2)	156/356	1.2 (0.9–1.6)
Insurance status				
Private	311/407	Ref	182/407	Ref
Medicare	236/332	0.8 (0.5–1.3)	131/332	1.1 (0.7–1.6)
Medicaid	120/163	1.2 (0.7–2.2)	75/163	1.2 (0.7–1.9)
VA/TRICARE/IHS/Other	46/70	1.8 (0.9–3.8)	34/70	0.6 (0.3–1.1)
Uninsured	42/60	0.9 (0.4–1.7)	24/60	0.9 (0.5–1.7)
Education				
High school education or less	156/217	Ref	79/217	Ref
Some college	294/408	1.2 (0.8–1.9)	186/408	1.2 (0.8–1.8)
College degree	192/259	1.5 (0.9–2.4)	115/259	1.0 (0.7–1.6)
Graduate education or higher	122/161	1.5 (0.8–2.7)	73/161	1.1 (0.7–1.8)
Income				
< $20,000	105/156	Ref	72/156	Ref
$20,000–$39,999	162/247	0.8 (0.5–1.4)	107/247	1.0 (0.6–1.6)
$40,000–$59,999	171/219	1.7 (0.9–3.0)	87/219	0.9 (0.6–1.5)
$60,000–$79,999	119/151	1.5 (0.8–3.0)	63/151	1.0 (0.6–1.7)
$80,000–$99,999	68/89	1.2 (0.6–2.6)	40/89	1.2 (0.6–2.3)
$100,000 +	139/183	1.2 (0.6–2.3)	84/183	1.2 (0.7–2.0)
General health status	2.7 (0.9)	0.9 (0.7–1.1)	2.9 (1.0)	1.1 (0.9–1.2)

Bold indicates statistical significance. Both models were estimated using multivariable logistic regression with adequate fit–area under ROC curves were 0.78 and 0.71, respectively. Values in the confidence intervals that would be rounded to 1 (i.e., 0.99) but are not significant were not rounded. Higher general health scores indicate worse health

**Table 4:** Correlates of FIT kit return preferences

	Mail v. Not (Ref.)		In-person Pharmacy v. Not (Ref.)		In-person PCP v. Not (Ref.)	
	*n*/*N* or mean (SD)	OR (CI_95%_)	*n*/*N* or mean (SD)	OR (CI_95%_)	*n*/*N* or mean (SD)	OR (CI_95%_)
Rurality						
Not Rural	583/731	Ref	246/731	Ref	167/731	Ref
Rural	257/314	1.1 (0.7–1.6)	73/314	**0.7 (0.5–0.9)**	68/314	1.1 (0.7–1.6)
Pharmacy quality scale	17.2 (5.1)	1.0 (0.9–1.0)	17.7 (4.8)	**1.0 (1.0–1.1)**	17.3 (4.9)	1.0 (0.9–1.0)
Usual method used to pick up prescriptions						
Mailed by pharmacy	186/225	Ref	55/225	Ref	50/225	Ref
In-person	654/820	0.9 (0.6–1.3)	264/820	**1.7 (1.1–2.4)**	185/820	1.0 (0.7–1.5)
Type of pharmacy most frequented						
A pharmacy in a clinic/hospital	77/90	Ref	27/90	Ref	27/90	Ref
A retail chain pharmacy	403/515	0.6 (0.3–1.1)	175/515	1.3 (0.7 – 2.2)	113/515	0.7 (0.4 – 1.3)
A pharmacy in a grocery store	134/164	0.7 (0.3–1.6)	44/164	0.9 (0.5–1.7)	35/164	0.7 (0.4–1.1)
A pharmacy in a department store or wholesaler	139/166	0.8 (0.4–1.8)	46/166	1.0 (0.6–1.9)	41/166	0.9 (0.5–1.7)
A local independently owned pharmacy	87/110	0.6 (0.3–1.4)	27/110	0.9 (0.4–1.8)	19/110	**0.5 (0.2–0.9)**
Travel time to nearest provider	19.0 (14.2)	1.0 (0.9–1.0)	19.3 (16.9)	1.0 (0.9–1.0)	18.7 (17.3)	1.0 (0.9–1.0)
Age						
45–64	560/696	Ref	221/696	Ref	164/696	Ref
65–75	280/349	0.7 (0.4–1.1)	98/349	1.0 (0.7–1.5)	71/349	1.1 (0.7–1.7)
Gender						
Male	408/523	Ref	179/523	Ref	122/523	Ref
Female	432/522	1.4 (0.9–1.9)	140/522	**0.7 (0.5–0.9)**	113/522	1.0 (0.7–1.3)
Race						
White	630/770	Ref	209/770	Ref	156/770	Ref
Black	100/134	0.7 (0.4–1.1)	49/134	1.3 (0.9–2.0)	42/134	**1.8 (1.1–2.7)**
Asian	49/68	**0.5 (0.3–0.9)**	38/68	**3.3 (1.9–5.7)**	20/68	**2.2 (1.2–4.0)**
Multiracial or other race	61/73	1.4 (0.7–2.9)	23/73	1.0 (0.6–1.9)	17/73	0.8 (0.4–1.6)
Ethnicity						
Non-hispanic or latino/a	746/919	Ref	270/919	Ref	198/919	Ref
Hispanic or latino/a	94/126	**0.6 (0.4–0.9)**	49/126	**1.6 (1.0–2.4)**	37/126	**1.8 (1.1–2.8)**
Screening history						
Recent screening	566/689	Ref	198/689	Ref	150/689	Ref
No recent screening	274/356	0.7 (0.5–1.0)	121/356	1.3 (0.9–1.8)	85/356	1.1 (0.8–1.5)
Insurance status						
Private	336/407	Ref	128/407	Ref	85/407	Ref
Medicare	278/332	1.3 (0.8–2.2)	86/332	0.8 (0.5–1.3)	56/332	0.7 (0.4–1.1)
Medicaid	115/163	**0.6 (0.3–0.9)**	63/163	**1.6 (1.0–2.6)**	60/163	**2.0 (1.2–3.3)**
VA/TRICARE/IHS/Other	55/70	0.7 (0.3–1.4)	23/70	1.3 (0.7–2.5)	20/70	1.3 (0.7–2.5)
Uninsured	47/60	0.9 (0.4–1.8)	15/60	0.7 (0.4–1.4)	11/60	0.7 (0.3–1.5)
Education						
High school education or less	161/217	Ref	66/217	Ref	52/217	Ref
Some college	333/408	1.3 (0.8–2.0)	111/408	0.9 (0.6–1.3)	103/408	1.2 (0.8–1.8)
College degree	212/259	1.4 (0.8–2.3)	91/259	1.2 (0.7–1.8)	57/259	1.0 (0.6–1.6)
Graduate education or higher	134/161	1.4 (0.8–2.5)	51/161	1.0 (0.6–1.7)	23/161	0.6 (0.3–1.2)
Income						
< $20,000	121/156	Ref	42/156	Ref	47/156	Ref
$20,000–$39,999	187/247	0.7 (0.4–1.2)	83/247	**1.6 (1.0–2.7)**	63/247	1.0 (0.6–1.7)
$40,000–$59,999	180/219	1.0 (0.6–1.9)	64/219	1.3 (0.8–2.3)	52/219	0.9 (0.5–1.7)
$60,000–$79,999	120/151	1.0 (0.5–1.8)	48/151	1.2 (0.7–2.1)	27/151	0.7 (0.4–1.3)
$80,000–$99,999	76/89	1.2 (0.6–2.8)	27/89	1.3 (0.7–2.5)	16/89	0.6 (0.3–1.4)
$100,000 +	156/183	1.3 (0.9–2.6)	55/183	1.1 (0.6–2.1)	30/183	0.6 (0.3–1.2)
General health status	2.8 (0.9)	1.1 (0.9–1.3)	2.8 (1.0)	1.0 (0.9–1.2)	2.8 (0.9)	**0.8 (0.7–0.9)**

Bold indicates statistical significance. All models were estimated using multivariable logistic regression with adequate fit–area under ROC curves were each 0.67. Values in the confidence intervals that would be rounded to 1 (i.e., 0.99) but are not significant were not rounded. Values in the confidence intervals that would be rounded to 1 (i.e., 0.99) but are not significant were not rounded. Higher general health scores indicate worse health

## Results

### Differences in respondents’ pharmacy experiences by rurality

A larger proportion of rural respondents used local, independently owned pharmacies compared with non-rural respondents (20.4%, 6.3%, *p* < 0.001; Table 2). Further, rural respondents reported higher perceptions of pharmacy service quality for every quality indicator compared with non-rural respondents, including familiarity with the pharmacy team (*p* < 0.001), pharmacy team is sympathetic and reassuring (*p* = 0.001), pharmacy team responds promptly (*p* = 0.004), pharmacy team gives me personal attention (*p* < 0.001), trust in the pharmacy team (*p* = 0.002), and feeling safe with pharmacy team (*p* = 0.003). The remaining pharmacy experience items did not differ by rurality.

### Design of the PharmFIT™ program: step 1—learn about the PharmFIT™ program

**Fig. 2:**
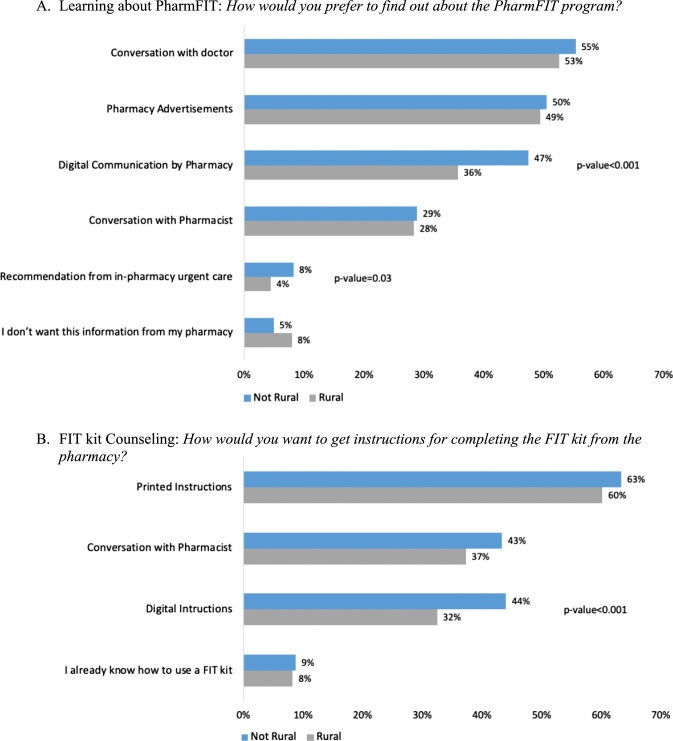
Patient Preferences **A** Learning about Pharm^FIT^ and **B** FIT kit Counseling

Respondents indicated preferences for a wide variety of ways to learn about the PharmFIT™ program with few differences by rurality (Fig. 2A). The most commonly preferred ways of learning about PharmFIT™ were conversations with their doctor (rural: 53%, non-rural: 55%), pharmacy advertisements (rural: 49%, non-rural: 50%), digital communication by the pharmacy (rural: 36%, non-rural: 47%), and conversations with their pharmacist (rural: 28%, non-rural: 29%). However, rural respondents reported preferring digital communication significantly less than non-rural respondents (*p* < 0.001). The most common way that respondents reported wanting their pharmacy to check their eligibility for FIT screening is through their doctor (rural: 74%, non-rural: 79%); however, nearly half of respondents also endorsed completing an eligibility survey at the pharmacy or in advance online (rural: 40%, non-rural: 45%). No differences by rurality were detected for FIT eligibility screening preferences (data not shown).

### Design of the PharmFIT™ program: step 2—Get the FIT kit from the pharmacy

Respondent preferences regarding receiving instructions for completing the FIT kit included printed instructions (rural: 60%, non-rural: 63%), conversation with their pharmacist (rural: 37%, non-rural: 43%), and digital instructions (rural: 32%, non-rural: 44%) (Fig. 2B). Significantly fewer rural respondents endorsed digital instructions than non-rural patients (*p* < 0.001). When asked to report preferences regarding the location of FIT kit counseling, most respondents endorsed in-person counseling (rural: 77%, non-rural: 77%). Most respondents who reported preferring in-person counseling also wanted at least a semi-private space to talk about CRC screening, with rural respondents requesting a private space less frequently (rural: 46%, non-rural: 52%). Digital counseling over the phone or videoconferencing was also endorsed frequently, but less so by rural respondents (rural: 41%, non-rural: 49%; *p* = 0.02).

In the multivariable logistic regression models, respondents who usually picked up their prescriptions in-person were more likely to want to pick up their FIT kit in-person at the pharmacy than those who usually received their prescriptions in the mail (OR 7.7, CI_95%_ 5.3–11.2; Table 3). Additionally, respondents who reported usually using a retail chain pharmacy (OR 3.0, CI_95%_ 1.7–5.4) or local independent pharmacy (OR 2.5, CI_95%_ 1.2–5.2), in comparison to a hospital or clinic pharmacy, were both more likely to want to pick up their FIT kit in-person.

In contrast, rural respondents (OR 0.7, CI_95%_ 0.5–0.9) and respondents who usually pick up their prescriptions at the pharmacy (OR 0.2, CI_95%_ 0.1–0.3) were less likely to want their FIT kit mailed to them (Table 3). Respondents who used any pharmacy types other than hospital or clinic pharmacies were less likely to prefer mailed FIT kits. However, Black respondents (OR 1.5, CI_95%_ 1.0–2.3) and multi-racial respondents (OR 2.3, CI_95%_ 1.3–4.0) were more likely to prefer their FIT kit delivered by mail than White respondents.

### Design of the PharmFIT™ program: step 3—complete and return the FIT kit

When asked about how they would like to be reminded to return their FIT kit, a vast majority of respondents reported digital communications, such as a phone call, text, or email. However, rural respondents reported these methods significantly less than non-rural respondents (rural: 82%, non-rural: 90%: *p* = 0.001). A reminder from their doctor (rural: 20%, non-rural: 24%), a reminder from the pharmacist next time they pick up a prescription (rural: 11%, non-rural: 11%), and a mailed letter (rural: 14%, non-rural: 18%) were endorsed much less frequently and did not differ based on a respondent’s rurality.

Respondents who usually picked their prescriptions up in-person were more likely to want to return their kit in-person to the pharmacy (OR 1.7, CI_95%_ 1.1–2.4; Table 4). However, rural respondents were less likely to endorse returning their kit in-person to the pharmacy than non-rural respondents (OR 0.7, CI_95%_ 0.5–0.9). Rurality was not significantly associated with return method in either the mailed or return in-person to their PCP models. However, respondents who typically used local independent pharmacies were less likely to want to return their FIT kit to their PCP in-person than those who used a hospital or clinic pharmacy (OR 0.5, CI_95%_ 0.2–0).

**Fig. 3:**
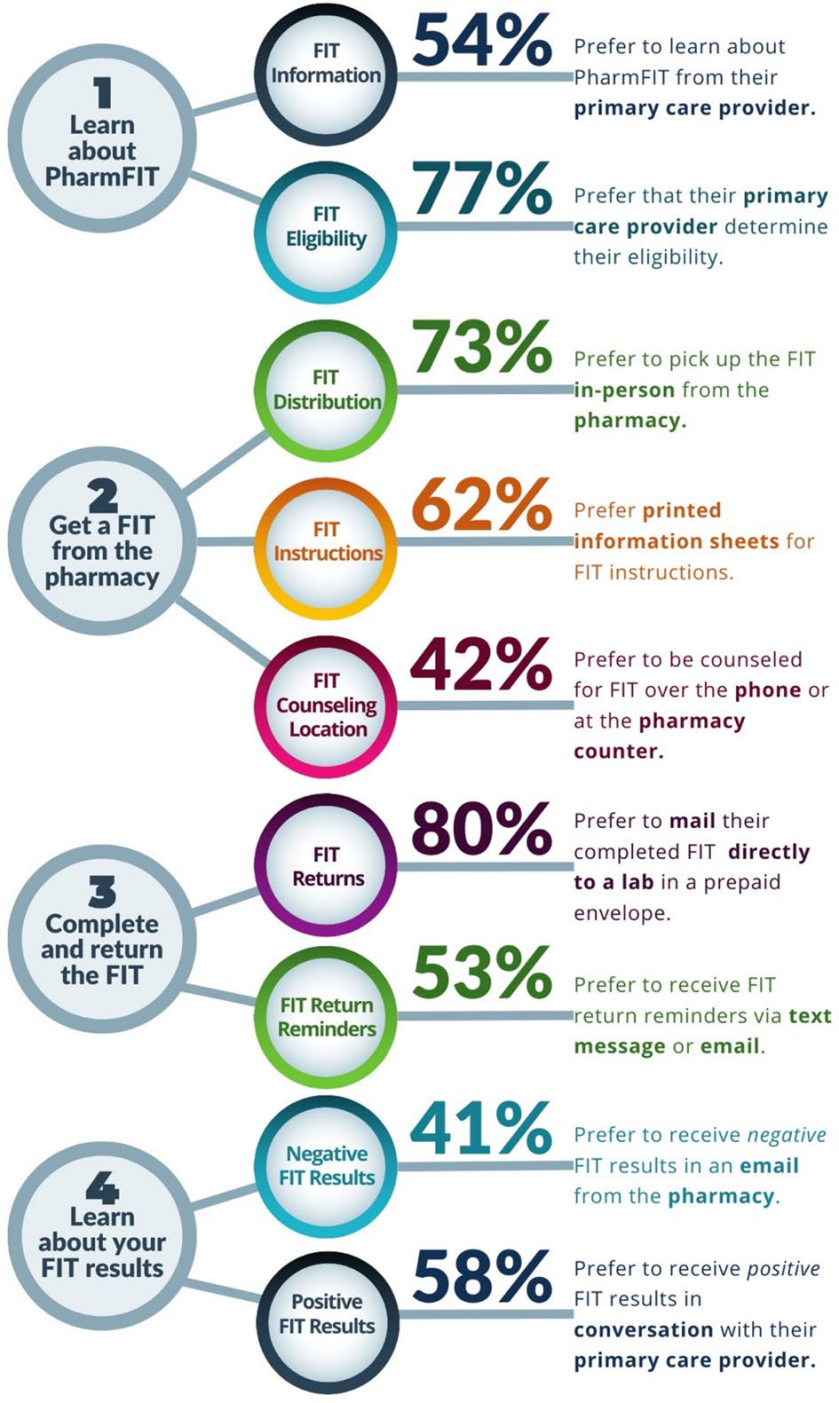
The Ideal Pharm^FIT^ Intervention from the Patient Perspective. Step 4 is not analyzed in this manuscript

Figure 3 depicts the most commonly reported design preferences for each step as an idealized model for the PharmFIT™ program, regardless of rurality.

## Discussion

This is the first national survey of U.S. adults ages 45–75 to assess preferences for the design of a pharmacy-based FIT distribution program called PharmFIT™. We oversampled people living in rural zip codes because studies have found unique barriers to CRC screening [30] and lower rates of screening compared to urban counterparts, particularly at the state level [31–33]. There were some notable differences in pharmacy use patterns across rural and non-rural populations; compared with non-rural zip codes, people in rural zip codes more often identified an independent pharmacy as their primary pharmacy, went more often in-person to pick up prescriptions and reported a more personal, trusting relationship with their pharmacist. We also found that preferences around two key design features—how to get a FIT and how to return a completed FIT—were also driven by pharmacy use patterns and perceptions of pharmacy quality.

Rural communities, particularly when intersecting with persistent poverty, are disproportionately impacted by screen-preventable cancers, like CRC [34–36]. Cancer screening services are traditionally delivered as part of a medical visit, which depends on access and, importantly, visit time to address prevention. Even when a person has access to primary care, limited visit time for preventive services is often a barrier to addressing preventive services [37]. The time needed to address recommended preventive services vastly outsizes the amount of time spent on preventive services [38, 39]. There have been calls, over the years, to “share the care,” expanding the medical neighborhood for delivery of some healthcare services [40]. Interventions conducted outside of medical visits, such as mailed FIT outreach, have the potential to help to bridge this gap. However, most FIT outreach programs and interventions to support screening in rural residents and populations with low income have been delivered in primary care settings [13]. Community pharmacies providing healthcare to their local communities may serve as an additional option for FIT distribution.

People live and work in places that may be very different from where they access primary care services, which may partially explain lower rates of CRC screening in rural versus urban counties [41, 42]. PharmFIT™ aims to address social barriers for completing CRC screening by making use of a venue that is often overlooked for cancer prevention services. Pharmacies are geographically more evenly distributed than primary care facilities and, as such, are the most accessible healthcare setting in the US; 97% of Americans live within 10 miles of a pharmacy and about one-third of pharmacies serve rural or low-income communities [14]. Patients visit community pharmacies at least two to three times as often as their physician’s office [15]. Over the last several decades, community pharmacy practice has increasingly focused on delivery of patient care services, including preventive care services [16]. Today, the average community pharmacist today spends about 10% of their time providing patient care services not associated with prescription drug dispensing, such as targeted medication reviews focused on addressing drug therapy problems and closing gaps in care [43]. The COVID-19 pandemic further highlighted the critical role that community pharmacists play in delivering point-of-care testing to economically and geographically underserved populations [44]. Community pharmacies are, therefore, a logical resource to explore for distribution of CRC screening services.

There are few reported studies of FIT distribution through community pharmacies. Two US studies, conducted in California and Connecticut, used different designs. Potter et al. reported in 2010 a small pilot study comparing of two pharmacy-based CRC screening approaches. Participants were recruited from among patients visiting the pharmacy during a flu vaccination campaign. Participants who received FITs did so in-person and clinical test results were managed and followed up by the lead author’s clinical department. The authors do not report the number of pharmacy patients approached to participate in the study; however, in terms of response, this study was successful; nearly 60% of the participants receiving a FIT completed it. In the second study, from Holle (2019), participants were recruited by a variety of methods: flyers in the pharmacy and attached to prescription bags, word-of-mouth, and in a local television segment. Delivery of the FITs occurred by referral to the pharmacy after enrolled participants completed a survey. It is not clear how patient results were clinically managed. This approach did not appear efficient; only 5% (16/312) of patients approached agreed to be in the study and only 4 ultimately completed a FIT. Our survey results revealed that PharmFIT™ design preferences may be related to how a person has historically used their pharmacy. For example, those who typically pick up their prescriptions in-person also tended to prefer in-person pick-up of a FIT at the pharmacy. The implication of these findings, coupled with the variable response to the two previously tested models, is that a successful PharmFIT™ program should be adapted to the local context, including pharmacy patient use patterns.

Multi-level, multi-component interventions are more successful in promoting CRC screening than single-level, single-component programs [10, 45]. Further, certain interventions have a greater impact on screening uptake. In a meta-analysis of clinical interventions for CRC screening, Dougherty, et al., showed that mailed FIT outreach was the single most impactful CRC screening intervention. When combined with other interventions, such as provider education or patient navigation, the impact of mailed FIT outreach was greater than single-component interventions (summary RD, 7%; CI_95%_, 3–11%).[10]. PharmFIT™ may require interventions at the pharmacy and physician levels; respondents clearly indicated a preference for physician involvement in certain key components of PharmFIT™ delivery. When asked about FIT eligibility determination, more than three quarters reported preferring physician involvement. However, because the response options were “check all that apply,” we were able to see that nearly half also found completing a survey from their pharmacist to determine eligibility acceptable. The components of PharmFIT™ can be accomplished in multiple, reasonable ways. In this case, if a patient has a close, trusting relationship with their pharmacist, as rural patients stated that they had physician involvement in PharmFIT™ may only need to be minimal, but a paired, physician-centered component of PharmFIT™ may be important for some patients to find it acceptable.

### Implications

The survey response revealed several important overarching implications for the design of a PharmFIT™ program. First, adaptability around the core functions of PharmFIT™ will be critical for both program effectiveness, in terms of CRC screening uptake, and program acceptability. Prior to implementing PharmFIT™, an assessment of the local context, including rurality and pharmacy type penetration (e.g., more locally owned independent pharmacies vs more chain pharmacies), should be conducted to specify the intervention such that it is most likely to meet the needs of the target population. In development of several pilot tests of PharmFIT™, the results of which will be reported elsewhere, we looked at these data, as well as additional formative data, to inform the design. To contextualize the design, we included a process mapping exercise with our pharmacy and primary care partners to adapt the intervention [46]. This exercise resulted in three distinct adaptations of PharmFIT™. [47] Second, PharmFIT™ services need to be wrapped around primary care. While respondents were quite supportive of the delivery of and counseling around CRC screening being conducted in the pharmacy setting, other aspects were more tied to primary care. This, too, could take multiple forms and should be adaptable. For example, primary care practices could generate lists of patients not up-to-date with CRC screening for pharmacists to contact and encourage FIT pick-up at the pharmacy. Other models could include direct referral from the provider to the pharmacy for screening or, similar to Potter [18], delivery in conjunction with campaigns targeting other preventive services, such as influenza vaccination.

### Strengths and limitations

This study has limitations. We used purposive sampling based on 2010 Census and oversampled rural residents, so our sample may not be representative of the general U.S. population eligible for FIT. Additionally, nearly two-thirds of respondents had some previous experience with CRC screening; it is possible that respondents without screening experience may have different preferences. Finally, PharmFIT™ was presented as a hypothetical program; while we provided explanations and visuals for each component of the program, it is possible that respondents’ preferences may be different after experiencing an implemented PharmFIT™ program at their pharmacy. However, this study also has strengths. It is, to our knowledge, the first national survey to directly assess preferences around the design of a pharmacy-based FIT distribution program. We did not purchase demographic data of ineligible panel members or those who did not take the study screener and, thus, were unable to compare demographic characteristics of survey participants to non-responders. However, all states are represented in our respondent pool, as well as Washington, DC, and Puerto Rico. Additionally, in over-sampling for rural residents, we achieved our aim of comparing outcomes across rural and non-rural residents. Further, our survey instrument was well-conceived; we used several validated scales and previously tested items to measure specific domains and cognitively tested other items to ensure appropriate understanding.

## Conclusion

The survey responses revealed differences in design preferences across certain subgroups; these differences support the need for an adaptable design that takes into account the local context and patient population. In addition to this national survey of patients, we are evaluating attitudes and preferences toward PharmFIT™ in a national survey of community pharmacists. Additionally, we have completed three small pilots of PharmFIT™ using differentiated delivery models [46, 47]. Our next step is to test a large-scale PharmFIT™ intervention in multiple community pharmacies. Through this experiment, we will be able to determine its impact on population uptake of FIT for colorectal cancer screening and its implementation, including cost.

### Supplementary Information

Below is the link to the electronic supplementary material.

Supplementary file1 (PDF 73 KB)

## Data Availability

The datasets generated during and/or analyzed during the current study are not publicly available due to ongoing analyses but are available from the corresponding author on reasonable request.
