# Supplementary material for: Patient preferences for the design of a pharmacy-based colorectal cancer screening program
Source: Cancer Causes Control. 2023 Apr 19;34(Suppl 1):99–112. doi: 10.1007/s10552-023-01687-x (PMC10113122; doi:10.1007/s10552-023-01687-x)
Supplement: Supplementary file 1 — Supplementary file1 (PDF 73 KB) [file 10552_2023_1687_MOESM1_ESM.pdf]

Qualtrics numbers

gc=1 - good complete responses (You'll notice that the gc=1 count is 1274 in the report, but we ended with 1045 good completes. This is because some responses that were initially counted as good completes were removed for data quality issues upon review of the data. In this case, 229 were removed.)

gc=2 - Terminated for not meeting one of the screening requirements – 2,128

gc=3 - Terminated for an overquota – 2085 - over our quotas for race/ethnicity, gender and/or rurality

gc=4 - Terminated for speeding - 50

Response Rate Using Response Rate 4:

$$\frac{I+P}{(I+P)+(R+NC+O)+e(UH+UO)} = \%$$

	**Definition**	**# in our sample**
I	complete interview. An eligible case that completed the interview	1,045
P	Partial interview. Any eligible who has answered all applicable questions (until Section H) but not completed.	229
R	Refusal or break-off. An eligible case that refuses to do the interview or breaks off of the interview.	50
NC	Non-contact. An eligible case that was not contacted. We do not know whether cases not contacted were eligible.	N/A
O	Other	N/A
UH	Unknown household. A household not reached whose eligibility is unknown.	N/A
UO	Unknown, other. A case not reached whose eligibility is unknown. In our sample, same as above.	2,085
e	Estimated proportion of cases of unknown eligibility in the sample. In estimating e, one must be guided by the best available scientific information on what share eligible cases make up among the unknown cases and one must not select a proportion in order to boost the response rate. $\frac{(1{,}045 + 229)}{(1{,}045 + 229) + (2{,}128 + 50)}$	.369

$$\frac{(1{,}045+229)}{(1{,}045+229)+(50)+.369(2{,}085)} = .623 = \mathbf{62.3\%}$$
